# Comparison of Time to Clinical Improvement With vs Without Remdesivir Treatment in Hospitalized Patients With COVID-19

**DOI:** 10.1001/jamanetworkopen.2021.3071

**Published:** 2021-03-24

**Authors:** Brian T. Garibaldi, Kunbo Wang, Matthew L. Robinson, Scott L. Zeger, Karen Bandeen-Roche, Mei-Cheng Wang, G. Caleb Alexander, Amita Gupta, Robert Bollinger, Yanxun Xu

**Affiliations:** 1 Division of Pulmonary and Critical Care Medicine, Johns Hopkins University School of Medicine, Baltimore, Maryland; 2 Department of Applied Mathematics and Statistics, Johns Hopkins University, Baltimore, Maryland; 3 Division of Infectious Disease, Johns Hopkins University School of Medicine, Baltimore, Maryland; 4 Division of Biostatistics, Johns Hopkins Bloomberg School of Public Health, Baltimore, Maryland; 5 Center for Drug Safety and Effectiveness, Johns Hopkins Bloomberg School of Public Health, Baltimore, Maryland; 6 Division of Biostatistics and Bioinformatics at The Sidney Kimmel Comprehensive Cancer Center, Johns Hopkins University School of Medicine, Baltimore, Maryland

## Abstract

**Importance:**

Clinical effectiveness data on remdesivir are urgently needed, especially among diverse populations and in combination with other therapies.

**Objective:**

To examine whether remdesivir administered with or without corticosteroids for treatment of coronavirus disease 2019 (COVID-19) is associated with more rapid clinical improvement in a racially/ethnically diverse population.

**Design, Setting, and Participants:**

This retrospective comparative effectiveness research study was conducted from March 4 to August 29, 2020, in a 5-hospital health system in the Baltimore, Maryland, and Washington, DC, area. Of 2483 individuals with confirmed severe acute respiratory syndrome coronavirus 2 infection assessed by polymerase chain reaction, those who received remdesivir were matched to infected individuals who did not receive remdesivir using time-invariant covariates (age, sex, race/ethnicity, Charlson Comorbidity Index, body mass index, and do-not-resuscitate or do-not-intubate orders) and time-dependent covariates (ratio of peripheral blood oxygen saturation to fraction of inspired oxygen, blood pressure, pulse, temperature, respiratory rate, C-reactive protein level, complete white blood cell count, lymphocyte count, albumin level, alanine aminotransferase level, glomerular filtration rate, dimerized plasmin fragment D [D-dimer] level, and oxygen device). An individual in the remdesivir group with *k* days of treatment was matched to a control patient who stayed in the hospital at least *k* days (5 days maximum) beyond the matching day.

**Exposures:**

Remdesivir treatment with or without corticosteroid administration.

**Main Outcomes and Measures:**

The primary outcome was rate of clinical improvement (hospital discharge or decrease of 2 points on the World Health Organization severity score), and the secondary outcome, mortality at 28 days. An additional outcome was clinical improvement and time to death associated with combined remdesivir and corticosteroid treatment.

**Results:**

Of 2483 consecutive admissions, 342 individuals received remdesivir, 184 of whom also received corticosteroids and 158 of whom received remdesivir alone. For these 342 patients, the median age was 60 years (interquartile range, 46-69 years), 189 (55.3%) were men, and 276 (80.7%) self-identified as non-White race/ethnicity. Remdesivir recipients had a shorter time to clinical improvement than matched controls without remdesivir treatment (median, 5.0 days [interquartile range, 4.0-8.0 days] vs 7.0 days [interquartile range, 4.0-10.0 days]; adjusted hazard ratio, 1.47 [95% CI, 1.22-1.79]). Remdesivir recipients had a 28-day mortality rate of 7.7% (22 deaths) compared with 14.0% (40 deaths) among matched controls, but this difference was not statistically significant in the time-to-death analysis (adjusted hazard ratio, 0.70; 95% CI, 0.38-1.28). The addition of corticosteroids to remdesivir was not associated with a reduced hazard of death at 28 days (adjusted hazard ratio, 1.94; 95% CI, 0.67-5.57).

**Conclusions and Relevance:**

In this comparative effectiveness research study of adults hospitalized with COVID-19, receipt of remdesivir was associated with faster clinical improvement in a cohort of predominantly non-White patients. Remdesivir plus corticosteroid administration did not reduce the time to death compared with remdesivir administered alone.

## Key Points

**Question:**

Does time to clinical improvement or time to death differ among hospitalized patients treated with vs without remdesivir (alone or with corticosteroids) for coronavirus disease 2019 outside of a clinical trial?

**Findings:**

In this multicenter comparative effectiveness research study that included 2483 consecutive admissions with a high proportion of non-White individuals, treatment with remdesivir was associated with more rapid clinical improvement than no remdesivir receipt in propensity score–matched controls. The addition of corticosteroids to remdesivir was not associated with improved time to death.

**Meaning:**

Remdesivir administration is associated with more rapid clinical improvement than no remdesivir receipt among patients with coronavirus disease 2019.

## Teaser

This propensity score–matched multicenter comparative effectiveness research study that included a high proportion of non-White individuals assesses whether remdesivir administered alone or with corticosteroids is associated with time to clinical improvement or time to death in patients hospitalized with confirmed coronavirus disease 2019 (COVID-19).

## Introduction

The pandemic caused by severe acute respiratory syndrome coronavirus 2 (SARS-CoV-2) continues to progress, with more than 103 million global cases and more than 2.2 million deaths as of February 2, 2021.^1^ While the world awaits the distribution of effective vaccines, a number of pharmacologic agents have been studied for treatment of coronavirus disease 2019 (COVID-19), the syndrome caused by SARS-CoV-2. Remdesivir, a nucleotide analogue prodrug with in vitro effects against a broad array of RNA viruses,^2,3,4^ has received considerable attention.^3,5,6^ Clinical trials assessing the effectiveness of remdesivir have yielded conflicting results^7,8,9,10,11,12^; have not included a sufficient number of patients from groups most affected by COVID-19, such as Black or Latinx individuals^13^; and have not examined the coadministration of remdesivir with other drugs. The US Food and Drug Administration (FDA) approved the use of remdesivir for the treatment of hospitalized patients with COVID-19^14^ based mostly on the results of the Adaptive COVID-19 Treatment Trial (ACTT-1),^11^ whereas the World Health Organization (WHO) recommended against the use of remdesivir^15^ based on results from the Solidarity trial.^12^

In addition to uncertainty about efficacy, there are concerns that the demand for remdesivir will outpace supply, raising calls for a transparent and just distribution process.^16^ Black individuals and Latinx individuals have mortality rates that are 2.4 and 1.5 times as high as White individuals, respectively.^17^ However, only 11% to 20% of participants in remdesivir clinical trials were Black patients, and only 17% to 23% were Latinx patients.^9,10^ Because underrepresented minority groups shoulder a disproportionate burden of morbidity and mortality from COVID-19,^18^ it is critical that we understand the effects of remdesivir in these populations.

The UK-based RECOVERY (Randomised Evaluation of COVID-19 Therapy) trial showed that dexamethasone administration compared with placebo led to a significant reduction in mortality rate (22.9% vs 25.7%).^19^ Trials examining the benefit of different corticosteroids were stopped early after the results of RECOVERY led to corticosteroids being considered the standard of care.^20,21^ Whether there is additional benefit from using both remdesivir and corticosteroids requires further evaluation. We used time-dependent propensity score matching to examine the association of remdesivir administration with clinical improvement in patients admitted to our 5-hospital health system from March 4 through August 29, 2020.

## Methods

### Study Design and Participants

This comparative effectiveness study was conducted at 5 hospitals (Johns Hopkins Hospital, Baltimore, Maryland; Bayview Hospital, Baltimore, Maryland; Howard County General Hospital, Columbia, Maryland; Suburban Hospital, Bethesda, Maryland; and Sibley Hospital, Washington, DC) that comprise the Johns Hopkins Medicine System, with 2513 beds serving approximately 7 million persons. All patients consecutively admitted with SARS-CoV-2 infection confirmed by polymerase chain reaction testing from nasal, oropharyngeal, or bronchoalveolar lavage samples between March 4 and August 29, 2020, were eligible. This study was conducted according to the International Society for Pharmacoeconomics and Outcomes Research (ISPOR) reporting guideline for comparative effectiveness research.^22^ The hospital institutional review boards approved this study as minimal risk and waived informed consent requirements. No one received compensation or was offered any incentive for participating in this study.

### Data Collection

The data used were part of JH-CROWN: The COVID Precision Medicine Analytics Platform Registry.^23,24^ Some patients were included in a previous description of the cohort.^24^ Race and ethnicity were derived from patient-reported values in the electronic health record.

### Eligibility Criteria and Administration of Remdesivir

The health system remdesivir policy followed the guidance from the US FDA initial Emergency Use Authorization.^25^ Patients prescribed remdesivir were required to have significant illness (oxygen saturation ≤94% breathing ambient air or requiring supplemental oxygen, mechanical ventilation, or extracorporeal membrane oxygenation) and have an alanine aminotransferase level less than 5 times the upper reference limit. Patients who were intubated at the end of a 5-day treatment could receive an additional 5 days if they had not improved. Treatment was stopped if the alanine aminotransferase level increased more than 5 times the upper reference limit or if there were other signs or symptoms of liver toxicity. Physicians were encouraged to stop remdesivir if the creatinine clearance decreased by more than 50% with no alternative explanation. Patients who received at least 1 dose of remdesivir outside of a clinical trial were considered to have been treated with remdesivir.

### Outcomes

The primary outcome of interest was time to clinical improvement from the start of remdesivir, a composite outcome defined as discharged alive from the hospital without worsening of WHO disease severity score or at least a 2-point decrease in WHO severity score during hospitalization within 28 days or maximum follow-up.^26^ Failure of clinical improvement was censored at the last day of follow-up or 28 days, whichever came first. Death was also censored at 28 days. The secondary outcome was time to death from the first remdesivir treatment day. Patients who were discharged alive were censored at 28 days to account for death and discharge being competing risks, as previously described.^27^ An additional secondary outcome was the time to clinical improvement or to death following administration of both corticosteroids and remdesivir.

### Statistical Analysis

Cox proportional hazards regression models were applied to estimate the association between remdesivir treatment and outcomes of interest. A set of demographic characteristics, clinical variables (including admission hospital), and laboratory results were included in the Cox proportional hazards regression models based on clinical interest and knowledge (Table). To account for the nonrandomized assignments of remdesivir and different timing of initial administration, we used time-dependent propensity score matching to create pairs of individuals, with 1 patient treated and the other patient being the most similar patient eligible for treatment at the time of initiation. Beginning from day 0, sequential 1:1 greedy matching without replacement was conducted. Propensity scores were calculated from a time-dependent Cox proportional hazards regression model using the time to first receipt of remdesivir as the outcome, in which the propensity score at a given hospitalization day is the hazard of exposure to remdesivir treatment on that day. Analyses were performed on the matched sets.^28,29^ Patients were included in matching if their admission dates were later than the earliest admission date (April 27, 2020) among patients in the remdesivir treatment group. In addition, a time constraint was imposed so that a patient in the remdesivir group with *k* days of treatment was matched to a patient in the control group who stayed in the hospital at least *k* days (5 days maximum) beyond the matching day. The last condition avoided matches in which the control patient was healthy enough to be discharged soon after the matched day because those patients would not be similar to patients who started remdesivir (eMethods in the Supplement). Data were analyzed using R, version 3.6.2 (R Foundation for Statistical Computing).

**Table.** Characteristics of Patients Before and After Propensity Score Matching

Characteristic	All patients^a^			Propensity score–matched patients^b^		
	All remdesivir (n = 342)	All control (n = 1957)	Absolute standardized difference	Matched remdesivir (n = 285)	Matched control (n = 285)	Absolute standardized difference
Demographic characteristics						
Sex, No. (%)						
Male	189 (55.3)	1004 (51.3)	0.079	160 (56.1)	158 (55.4)	0.014
Female	153 (44.7)	953 (48.7)		125 (43.9)	127 (44.6)	
Race/ethnicity, No. (%)						
Black	124 (36.3)	715 (36.5)	0.006	95 (33.3)	100 (35.1)	0.037
Latinx	114 (33.3)	519 (26.5)	0.149	98 (34.4)	86 (30.2)	0.090
White	66 (19.3)	534 (27.3)	0.190	59 (20.7)	66 (23.2)	0.059
Other^c^	38 (11.1)	189 (9.7)	0.048	33 (11.6)	33 (11.6)	0
Age, median (IQR), y	60 (46-69)	60 (44-74)	0.025	60 (48-70)	62 (51-75)	0.167
BMI, median (IQR)	30.1 (25.7-36.0)	28.2 (24.1-33.2)	0.225	29.8 (25.9-34.7)	29.6 (25.4-35.0)	0.070
DNR or DNI, No. (%)	61 (17.8)	435 (22.2)	0.110	59 (20.7)	69 (24.2)	0.084
Oxygen devices, No. (%)						
No supplemental oxygen	16 (4.7)	907 (46.3)	1.088	16 (5.6)	15 (5.3)	0.015
Nasal cannula or face mask	210 (61.4)	819 (41.8)	0.399	189 (66.3)	173 (60.7)	0.117
High-flow nasal cannula	60 (17.5)	79 (4.0)	0.446	38 (13.3)	50 (17.5)	0.117
Noninvasive positive-pressure ventilation	5 (1.5)	34 (1.7)	0.022	5 (1.8)	5 (1.8)	0
Mechanical ventilator	51 (14.9)	105 (5.4)	0.320	37 (13.0)	39 (13.7)	0.021
Vital signs, mean (SD)						
Temperature, °C	37.9 (0.9)	37.6 (0.9)	0.246	37.8 (0.8)	37.9 (0.9)	0.090
Pulse, beats/min	97.6 (18.0)	96.9 (19.5)	0.039	96.4 (19.6)	97.7 (18.7)	0.064
Systolic BP, mm Hg	109.1 (17.4)	110.1 (19.3)	0.057	106.2 (16.5)	107.6 (17.6)	0.086
Diastolic BP, mm Hg	60.9 (10.9)	61.2 (12.0)	0.025	58.7 (10.3)	58.3 (10.9)	0.040
Spo_2_:Fio_2_ ratio, mean (SD)	355.9 (115.3)	420.1 (108.6)	0.573	345.8 (108.1)	334.5 (122.6)	0.098
Laboratory results, mean (SD)						
Estimated glomerular filtration rate, mL/min/1.73 m^2^	81.5 (30.3)	77.4 (36.1)	0.124	85.9 (29.9)	79.7 (34.9)	0.192
C-reactive protein, mg/dL	11.1 (8.1)	8.8 (8.5)	0.285	11.5 (8.5)	12.1 (10.4)	0.055
Absolute lymphocyte count, /µL	1100 (600)	1300 (2000)	0.137	1100 (700)	1000 (600)	0.124
Platelet count, ×10^3^/µL	215 900 (91 200)	217 200 (93 300)	0.015	233 200 (102 700)	225 300 (98 300)	0.078
White blood cell count, cells/µL	7900 (5300)	8100 (6900)	0.037	8200 (6300)	8200 (4400)	0.000
Hemoglobin, g/dL	12.6 (2)	12.2 (2.2)	0.202	12.1 (1.9)	12 (2.3)	0.053
Albumin, g/dL	3.4 (0.6)	3.5 (0.6)	0.134	3.2 (0.6)	3.1 (0.6)	0.100
Alanine aminotransferase, U/L	44.7 (36.1)	50.4 (164.1)	0.047	44.5 (38.4)	41.8 (39.9)	0.068
D-dimer, µg/mL FEU	2 (4.2)	2.1 (4.2)	0.019	2.3 (4.7)	2.2 (4.1)	0.028
Past diagnoses, No. (%)^d^						
Hypertension	152 (44.4)	906 (46.3)	0.037	131 (46.0)	131 (46.0)	0
Coronary artery disease	92 (26.9)	599 (30.6)	0.082	82 (28.8)	107 (37.5)	0.187
Congestive heart failure	45 (13.2)	292 (14.9)	0.051	39 (13.7)	59 (20.7)	0.187
Chronic kidney disease	28 (8.2)	236 (12.1)	0.129	27 (9.5)	34 (11.9)	0.080
Diabetes	111 (32.5)	536 (27.4)	0.111	97 (34.0)	93 (32.6)	0.030
Asthma	29 (8.5)	164 (8.4)	0.004	23 (8.1)	22 (7.7)	0.013
COPD or chronic lung disease	59 (17.3)	307 (15.7)	0.042	49 (17.2)	39 (13.7)	0.097
Cancer	23 (6.7)	190 (9.7)	0.109	22 (7.7)	25 (8.8)	0.038
Liver disease	13 (3.8)	99 (5.1)	0.061	13 (4.6)	14 (4.9)	0.017
AIDS/HIV	3 (0.9)	26 (1.3)	0.043	2 (0.7)	5 (1.8)	0.096
Transplant	8 (2.3)	34 (1.7)	0.043	8 (2.8)	4 (1.4)	0.098
Charlson Comorbidity Index, score, No. (%)						
0	142 (41.5)	794 (40.6)	0.019	111 (38.9)	102 (35.8)	0.065
1-4	191 (55.8)	1089 (55.6)	0.004	166 (58.2)	176 (61.8)	0.072
≥5	9 (2.6)	74 (3.8)	0.065	8 (2.8)	7 (2.5)	0.022
Concomitant medications, No. (%)						
Hydroxychloroquine	3 (0.9)	399 (20.4)	0.667	3 (1.1)	7 (2.5)	0.107
Azithromycin	153 (44.7)	706 (36.1)	0.177	121 (42.5)	124 (43.5)	0.021
Dexamethasone	157 (45.9)	155 (7.9)	0.948	132 (46.3)	46 (16.1)	0.689
Prednisone	27 (7.9)	112 (5.7)	0.086	22 (7.7)	15 (5.3)	0.100
Methylprednisolone	20 (5.8)	88 (4.5)	0.061	14 (4.9)	23 (8.1)	0.128
Hydrocortisone	12 (3.5)	62 (3.2)	0.019	10 (3.5)	15 (5.3)	0.086
Heparin	292 (85.4)	1358 (69.4)	0.389	242 (84.9)	220 (77.2)	0.198

Abbreviations: BMI, body mass index (calculated as weight in kilograms divided by height in meters squared); BP, blood pressure; COPD, chronic obstructive pulmonary disease; D-dimer, dimerized plasmin fragment D; DNI, do not intubate; DNR, do not resuscitate; FEU, fibrinogen-equivalent unit; IQR, interquartile range; Spo_2_:Fio_2_, ratio of peripheral blood oxygen saturation to fraction of inspired oxygen.

SI conversion factors: To convert C-reactive protein to milligrams per liter, multiply by 10; lymphocyte count and white blood cell count to ×10^9^/L, by 0.001; platelet count to ×10^9^ per liter, by 1.0; hemoglobin to g/L, by 10.0; albumin to grams per liter, by 10; alanine aminotransferase to microkatals per liter, by 0.0167; D-dimer to nanomoles per liter, by 5.476.

^a^ Data shown are from day 0 of hospital admission.

^b^ Data shown are from the day of remdesivir treatment initiation.

^c^ Comprised non-White, non-Black, and non-Latinx patients.

^d^ Only the Charlson Comorbidity Index was used in the Cox proportional hazards models. Individual comorbidities are shown but were not used in matching.

#### Sensitivity Analyses

The analyses were repeated using the same propensity score–matching method but with the following conditions: (1) excluding remdesivir-treated patients who also received corticosteroids; (2) applying the ACTT-1^11^ inclusion/exclusion criteria to select qualified patients for matching; (3) decreasing the number of days that matched controls had to remain in the hospital after the matched day from a maximum of 5 days to either 4 or 3 days; and (4) categorizing clinical improvement using a 1-point decrease in WHO severity score instead of a 2-point decrease.

#### Combination of Remdesivir With Corticosteroids

We compared outcomes among patients who received corticosteroids plus remdesivir to patients who received remdesivir alone. Because the sample sizes for the 2 groups were similar and exposure to corticosteroids was time variant, marginal structural Cox proportional hazards regression models were used to adjust for the nonrandomized administration of corticosteroids and to analyze the association of corticosteroids with outcomes of interest in patients who had exposure to remdesivir. The same set of time-dependent and time-invariant covariates were included in the model. The inverse probability treatment weighting method was applied for parameter estimation^27,30^ (eMethods in the Supplement).

## Results

### Patients

**Figure 1.**
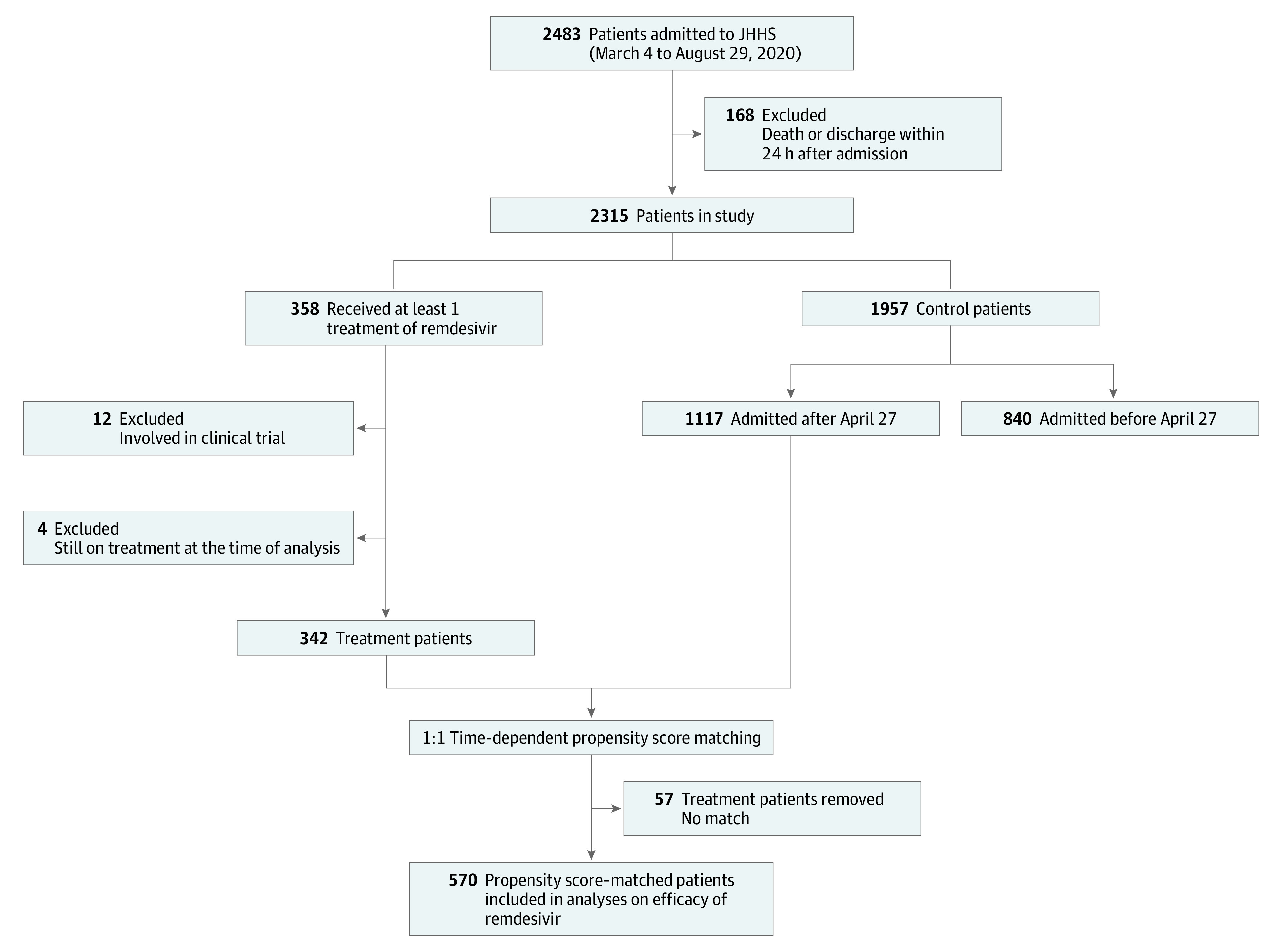
Description of Patients Included in the Analysis JHHS indicates the Johns Hopkins Health System.

Of 2483 patients admitted to the Johns Hopkins Health System with COVID-19 between March 4 and August 29, 2020, 184 were excluded. The reasons for exclusion included death or hospital discharge within 24 hours of admission (n = 168), participation in randomized clinical trials of remdesivir (n = 12), and being treated with remdesivir at the date of censoring (n = 4) (Figure 1). Among the remaining 2299 patients, 342 (14.9%) received remdesivir. Of those receiving remdesivir, the median age was 60 years (interquartile range [IQR], 46-69 years); 153 (44.7%) were women, and 189 (55.3%) were men; and 276 (80.7%) self-identified as non-White individuals. The percentage of patients who stopped treatment early was 2.9%. In addition, 303 patients (88.6%) received a 5-day course, 33 patients (9.6%) did not complete 5 days of treatment, and 6 patients (1.8%) received more than 5 days of treatment. The median time from admission to treatment initiation was 1.1 days (IQR, 0.8-2.4 days). After time-dependent propensity score matching, 285 patients (83.3%) in the remdesivir group were matched successfully and were selected for primary statistical analyses. Of those patients, 59 (20.7%) self-identified as being non-Latinx White, 95 (33.3%) non-Latinx Black, and 98 (34.4%) Latinx race/ethnicity. The Table shows the demographic and clinical characteristics of remdesivir recipients and controls before (day 0) and after (day 1 of treatment) propensity score matching.

### Time to Clinical Improvement

**Figure 2.**
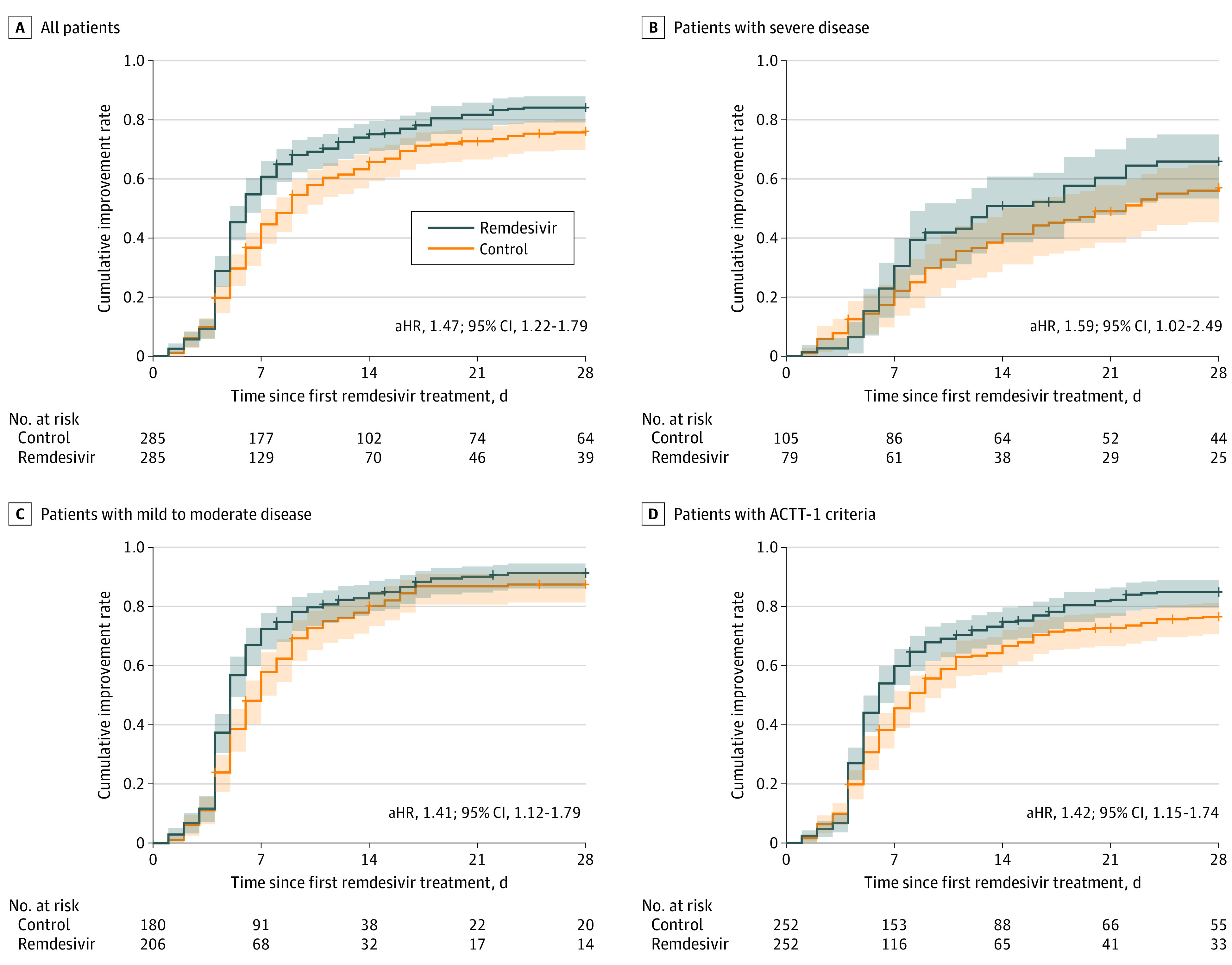
Time to Clinical Improvement Cumulative incidence curves for time to clinical improvement are shown for the entire remdesivir and matched control cohort (A); patients with severe disease (requiring high-flow nasal cannula, noninvasive ventilation, mechanical ventilation, extracorporeal membrane oxygenation, or vasopressors) (B); patients with mild to moderate disease (on ambient air or nasal cannula oxygen) (C); and patients who would have met Adaptive COVID-19 Treatment Trial 1 (ACTT-1) study criteria (D). aHR indicates adjusted hazard ratio.

Of 570 matched individuals (285 remdesivir and 285 matched controls), 236 (82.8%) patients who received remdesivir and 213 (74.7%) controls achieved clinical improvement before 28 days, with a median time to clinical improvement of 5.0 days (IQR, 4.0-8.0 days) for remdesivir recipients and 7.0 days (IQR, 4.0-10.0 days) for controls. In Cox proportional hazards regression models, remdesivir treatment was associated with significantly shortened time to clinical improvement (adjusted hazard ratio [aHR], 1.47 [95% CI, 1.22-1.79]) (Figure 2A). Patients treated with remdesivir and breathing ambient air or nasal cannula oxygen reached clinical improvement faster than matched controls (median, 5 days [IQR, 4.0-7.0 days] vs 6 days [IQR, 4.0-9.0 days]; aHR, 1.41 [95% CI, 1.12-1.79]); those with severe disease (requiring higher levels of respiratory support) also benefitted in the time to clinical improvement from remdesivir treatment (median, 8.0 days [IQR, 6.0-13.0 days] vs 9.0 days [IQR, 5.5-16.0 days]; aHR, 1.59 [95% CI, 1.02-2.49]) (Figure 2B and C).

### Time to Death

**Figure 3.**
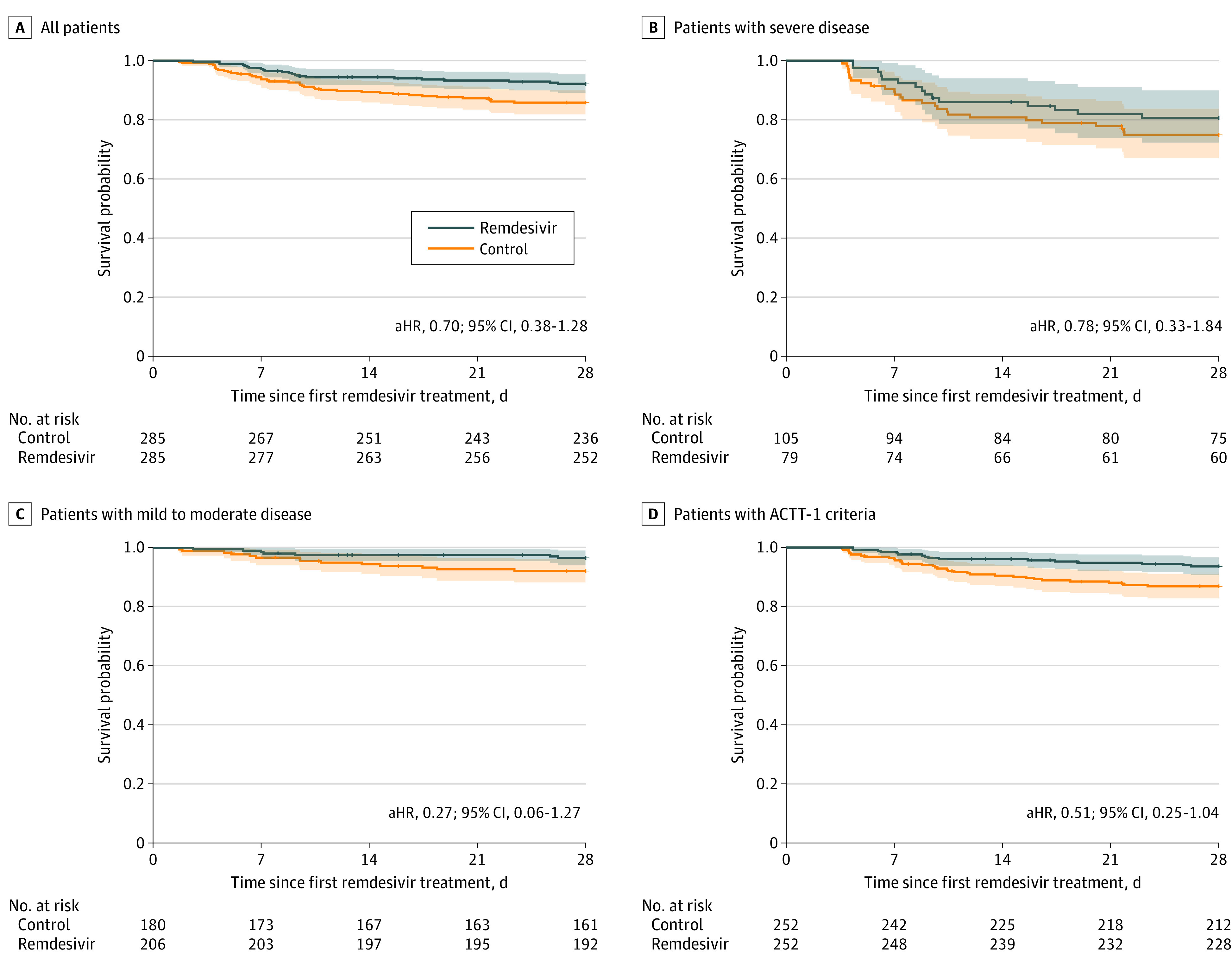
Patient Survival Kaplan-Meier survival curves are shown for the entire remdesivir and matched control cohort (A); patients with severe disease (requiring high-flow nasal cannula, noninvasive ventilation, mechanical ventilation, extracorporeal membrane oxygenation, or vasopressors) (B); patients with mild to moderate disease (on ambient air or nasal cannula oxygen) (C); and patients who would have met Adaptive COVID-19 Treatment Trial 1 (ACTT-1) study criteria (D). aHR indicates adjusted hazard ratio.

Remdesivir recipients had a 28-day mortality rate of 7.7% (22 deaths) compared with 14.0% (40 deaths) for matched controls, but this difference was not statistically significant in the time-to-death analysis (aHR, 0.70; 95% CI, 0.38-1.28) (Figure 3A). The median time to death was 8.6 days (IQR, 6.1-14.2 days) for remdesivir recipients and 8.2 days (IQR, 4.8-13.8 days) for controls. There was no significant mortality benefit associated with remdesivir treatment for patients with mild to moderate disease (aHR, 0.27; 95% CI, 0.06-1.27) or for patients with severe disease (aHR, 0.78; 95% CI, 0.33-1.84) (Figure 3B and C).

### Sensitivity Analyses

We considered only 306 remdesivir recipients who met inclusion criteria for ACTT-1 for the sensitivity analysis (eFigure 1 in the Supplement). Of them, 252 (82.4%) were successfully matched with control patients who also satisfied the criteria (eTable 1 in the Supplement). The median time to clinical improvement was 5.0 days (IQR, 4.0-8.0 days) for remdesivir recipients and 6.0 days (IQR, 4.0-10.0 days) for matched controls; remdesivir administration was associated with shortened time to clinical improvement (aHR, 1.42; 95% CI, 1.15-1.74) (Figure 2D). The mortality rate at 28 days was 6.3% (16 deaths) in the remdesivir group vs 13.1% (33 deaths) in controls, but this difference was not statistically significant (aHR, 0.51; 95% CI, 0.25-1.04) (Figure 3D). The median time to death was 9.1 days (IQR, 6.9-17.8 days) and 9.6 days (IQR, 6.7-14.7 days) for remdesivir recipients and control patients.

The results were sensitive to the requirement that controls be selected from among patients who remained hospitalized for the same duration of treatment as their matched counterpart (up to 5 days). Although remdesivir administration was still associated with a significant decrease in the time to clinical improvement if the requirement was reduced to 4 days (aHR, 1.25; 95% CI, 1.03-1.50), the estimated aHR decreased to 1.07 (95% CI, 0.89-1.28) if the required period of hospitalization was lowered to 3 days or less. This finding occurred because 87 patients in the control group had their event within 4 days of matching (78 discharged from the hospital, and 9 improved) compared with 26 patients in the treatment group (23 discharged, and 3 improved).

**Figure 4.**
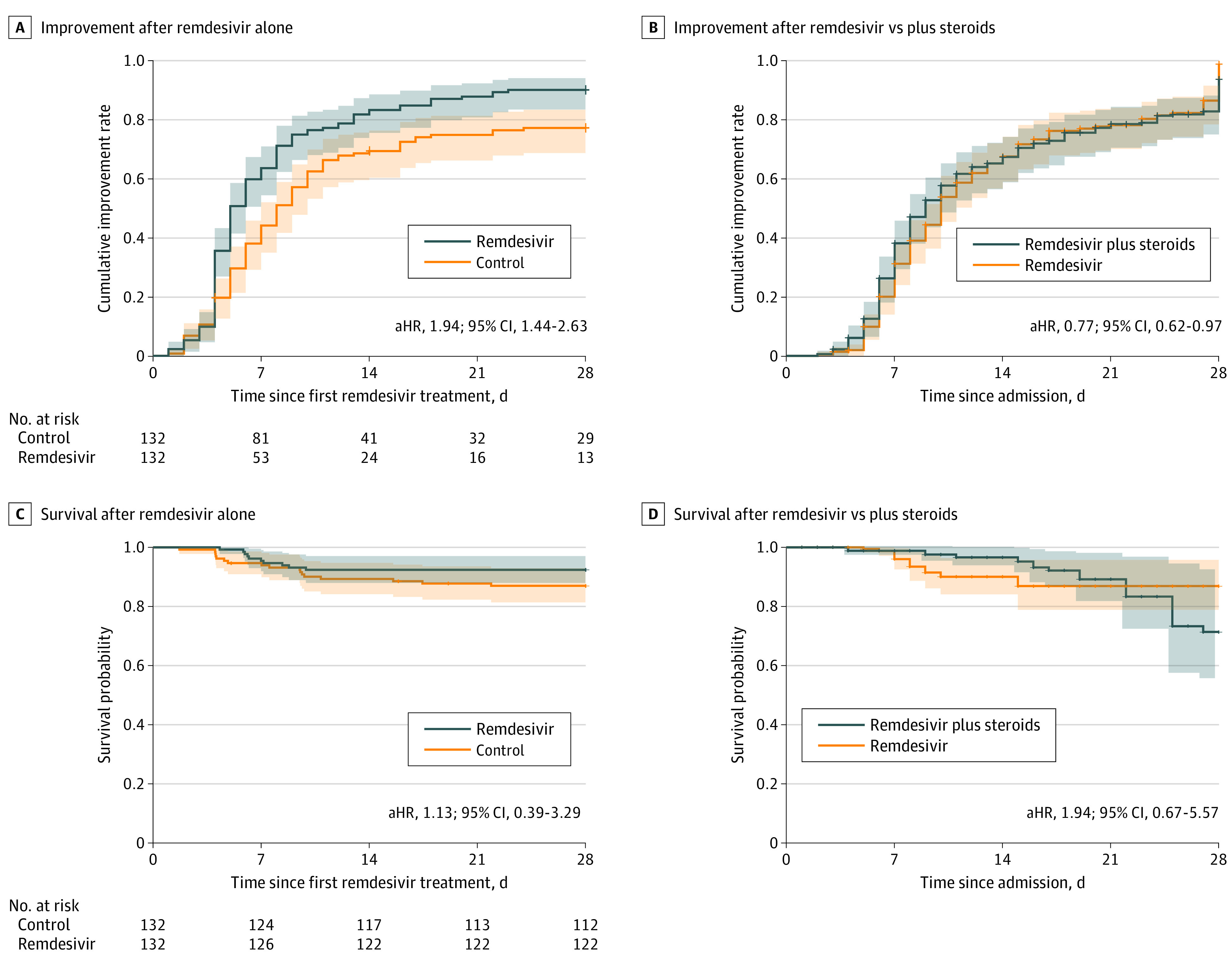
Clinical Improvement, Mortality, and Survival Rates by Receipt of Remdesivir Alone or Plus Corticosteroids A, Time to clinical improvement for patients who received remdesivir, excluding patients who also received corticosteroids. B, Adjusted cumulative improvement curve for patients who received remdesivir plus corticosteroids vs those who received remdesivir alone, adjusted using marginal structural Cox models. C, Kaplan-Meier survival curves for patients who received remdesivir, excluding patients who also received corticosteroids. D, Adjusted survival curve for patients who received remdesivir plus corticosteroids vs those who received remdesivir alone, adjusted using marginal structural Cox proportional hazards regression models. aHR indicates adjusted hazard ratio.

We repeated the time-to-improvement analyses using a 1-point improvement in the WHO severity score, and the results did not appreciably change (eMethods in the Supplement). We excluded 184 patients who received remdesivir plus corticosteroids (and their matched controls) from our initial analyses. Remdesivir alone still had a significant association with time to clinical improvement (aHR, 1.94; 95% CI, 1.44-2.63) (Figure 4A) and no significant association with mortality (aHR, 1.13; 95% CI, 0.39-3.29) (Figure 4C). These results remained unchanged if we only removed patients who received dexamethasone from the analysis (time to clinical improvement: aHR, 1.71 [95% CI, 1.30-2.26]; time to death: aHR, 1.32 [95% CI, 0.51-3.41]).

### Combination of Remdesivir With Corticosteroids

We compared 184 patients who received remdesivir plus corticosteroids with 158 patients who received remdesivir alone (eTable 2 and eFigure 2 in the Supplement). Combination therapy was associated with longer time to clinical improvement in the marginal structural Cox proportional hazards regression model (aHR, 0.77; 95% CI, 0.62-0.97) (Figure 4B). Unadjusted 28-day mortality rates were similar (15 deaths [8.2%] for the combination vs 10 deaths [6.3%] for remdesivir alone) (eTable 2 in the Supplement). The median time to death was increased for patients who received combination therapy (15.0 days; IQR, 9.0-21.0 days) compared with remdesivir alone (6.5 days; IQR, 6.0-7.8 days). A marginal structural Cox proportional hazards regression model did not show a significant reduction in the hazard of death for patients who received remdesivir and corticosteroids compared with remdesivir alone (aHR, 1.94; 95% CI, 0.67-5.57) (Figure 4D). In total, 68 individuals (37%) in the remdesivir and corticosteroid group had severe disease compared with 41 individuals (26%) in the remdesivir group. The sample sizes were too small to evaluate whether combined therapy was associated with benefits for patients with severe disease. If the analysis was restricted to only patients who received dexamethasone as the corticosteroid, the combination of dexamethasone and remdesivir was not associated with reduced mortality compared with remdesivir alone (aHR, 1.47; 95% CI, 0.46-4.67).

### Reasons for Stopping Remdesivir Early

Of 33 patients (9.6%) who did not complete at least 5 days of remdesivir administration, 21 were discharged from the hospital, and 2 died before completing treatment. For the remaining patients, treatment was stopped for the following reasons: increased levels of liver enzyme or bilirubin (n = 4), kidney failure of unclear cause (n = 2), nausea (n = 1), epistaxis and tachycardia (n = 1), neck and mouth itching (n = 1), and transition to comfort care (n = 1). The incidence of liver enzyme levels above 200 U/L (ie, alanine aminotransferase or aspartate aminotransferase; to convert to microkatals per liter, multiply by 0.0167), bilirubin levels above 2 mg/dL (to convert to micromoles per liter, multiply by 17.104), and estimated glomerular filtration rate less than 30 mL/min/1.73 m^2^ are shown in eTable 3 in the Supplement.

## Discussion

Optimal implementation of therapeutics to decrease COVID-19 morbidity and mortality is a global priority. Remdesivir has been the subject of controversy since ACTT-1, sponsored by the National Institutes of Health, showed a shortened time to clinical improvement, but the larger Solidarity study, sponsored by the WHO, did not show a mortality benefit. Although the results of Solidarity likely indicate that remdesivir alone does not have a robust mortality benefit for patients with COVID-19, remdesivir may still have an important role to play in reducing duration and severity of illness, both important outcomes when hospitals are overwhelmed with patients having COVID-19.^31^ In our retrospective multicenter study, receipt of remdesivir was associated with a significantly shorter time to clinical improvement. The mortality rate was lower in the remdesivir group, but the results were not statistically significant. The magnitude and direction of these associations were similar to those shown in ACTT-1.^32^

Our study included a much higher percentage of patients from underrepresented minority groups than previous remdesivir clinical trials. Approximately 80% of patients in our cohort were non-White individuals compared with 30% to 47% in clinical trials.^9,10,32^ Because underrepresented minority groups have shouldered a disproportionate burden during the COVID-19 pandemic but have not been widely represented in clinical trials,^13^ our results provide important evidence that receipt of remdesivir is associated with decreased time to clinical improvement in these populations.

The vast majority of patients in the remdesivir group received 5 days of therapy, which supports recommendations for an initial 5-day course for most patients. Although ACTT-1 used a 10-day treatment course, a comparison of 5 days vs 10 days showed similar efficacy,^9^ and an open-label study found that a 5-day course, but not a 10-day course, was associated with a significant improvement in disease severity.^10^ Our finding that a 5-day treatment course was associated with a clinical benefit is important, particularly given reports of both US and global shortages of remdesivir and the potential for even greater supply constraints moving forward.^33^

Our cohort included several groups of individuals who were not eligible to receive remdesivir in prior clinical trials, including 9 patients who were pregnant, 2 patients who were younger than 18 years, and 16 patients who showed evidence of chronic kidney disease at baseline. Inclusion of these patients was not associated with an increased incidence of significant adverse effects as evidenced by the small percentage of patients in our cohort who stopped treatment early (2.9%) compared with ACTT-1 (9%).

We also took into account the increased use of dexamethasone (and corticosteroids in general) after the RECOVERY trial.^19^ Removing patients who also received corticosteroids did not appreciably change the overall association of remdesivir with time to clinical improvement.

Although there was no significant reduction in the hazard of death for patients who received both remdesivir and corticosteroids, our low baseline mortality rate (6.3% for remdesivir-only patients) compared with the RECOVERY trial (26% for controls)^19^ and small numbers of events may have prevented us from detecting a benefit. The increased median time to death and the higher number of severely ill patients in the combination group suggest that the combination group was more severely ill and may have derived some benefit from the addition of corticosteroids that was not accounted for in the marginal structural model.

In ACTT-1, patients breathing ambient air or nasal cannula oxygen benefitted from treatment with remdesivir, whereas patients receiving higher levels of respiratory support, such as mechanical ventilation, did not benefit.^32^ Although our study showed a significant association of remdesivir with time to clinical improvement in both groups, the 95% CI in the severely ill group approached 1, suggesting perhaps a more robust benefit for patients with mild to moderate disease. By contrast, the RECOVERY trial showed that only patients receiving supplemental oxygen or additional respiratory support benefitted from dexamethasone, whereas patients breathing ambient air did not.^19^ Although ACTT-4 will provide some insight into the effectiveness of remdesivir combined with dexamethasone, the comparator group will be remdesivir in combination with baricitinib, and the results will not be available for several months.^34^ The combination of corticosteroids and remdesivir in specific target populations and the timing of coadministration warrant additional study.

### Limitations

Our study has limitations. The time-dependent propensity score method produced matched sets in which patients were very similar on measured confounders. However, there could be unmeasured variables that biased our treatment effect estimates. It is possible that there was a secular trend in the quality of COVID-19 patient care as our health system gained experience; thus, we limited our matched control group to the period when remdesivir was available. We required controls to remain hospitalized for the same amount of time as patients who received remdesivir treatment, not exceeding 5 days. This criterion was a prespecified analysis design to avoid bias from matching controls with different disease severity that could not be controlled for by the measurement confounders. However, our findings were sensitive to this restriction being reduced to 3 or fewer days. This protocol is consistent with ACTT-1, which excluded patients expected to be discharged from the hospital within 3 days of randomization.^32^ Among the unmatched control patients, 596 experienced their outcome within 5 days of admission, with 578 (97%) being discharged from the hospital. The matching assignment is therefore unlikely to bias the results toward the null hypothesis. We were likely underpowered to detect a mortality difference between patients receiving remdesivir and matched controls. There was a trend toward a reduction in mortality associated with remdesivir administration that was similar in magnitude to that shown in ACTT-1, but neither study result reached statistical significance.

## Conclusions

This study suggests that remdesivir was associated with a significant decrease in the time to clinical recovery among patients admitted to the hospital for treatment of COVID-19. These results provide further evidence that remdesivir may be effective in reducing the duration of COVID-19 illness, that a 5-day treatment course may be sufficient, and that patients with milder disease likely benefit most. The inclusion of a larger proportion of patients from underrepresented minority groups provides much-needed evidence suggesting the effectiveness of remdesivir administration in these groups. The combination of remdesivir and corticosteroids was not associated with reduced mortality, suggesting that additional studies assessing patients with COVID-19 are warranted.

## Notes

Supplement. **eMethods.** **eFigure 1.** Exclusion Details Under ACTT-1 Criteria **eFigure 2.** Timing of Remdesivir and Corticosteroid Coadministration **eTable 1.** Characteristics of Patients That Satisfied ACTT-1 Criteria, Before and After Propensity Score Matching **eTable 2.** Characteristics of Remdesivir Patients Stratified by Corticosteroid Use **eTable 3.** Adverse Events **eReferences.**
