# Supplementary material for: Comparison of Time to Clinical Improvement With vs Without Remdesivir Treatment in Hospitalized Patients With COVID-19
Source: JAMA Netw Open. 2021 Mar 24;4(3):e213071. doi: 10.1001/jamanetworkopen.2021.3071 (PMC7991975; doi:10.1001/jamanetworkopen.2021.3071)
Supplement: Supplement. — eMethods. eFigure 1. Exclusion Details Under ACTT-1 Criteria eFigure 2. Timing of Remdesivir and Corticosteroid Coadministration eTable 1. Characteristics of Patients That Satisfied ACTT-1 Criteria, Before and After Propensity Score Matching eTable 2. Characteristics of Remdesivir Patients Stratified by Corticosteroid Use eTable 3. Adverse Events eReferences. [file jamanetwopen-e213071-s001.pdf]

Supplementary Online Content

Garibaldi BT, Wang K, Robinson ML, et al. Comparison of time to clinical improvement with vs without remdesivir treatment in hospitalized patients with COVID-19. *JAMA Netw Open*. 2021;4(3):e213071. doi:10.1001/jamanetworkopen.2021.3071

eMethods.

eFigure 1. Exclusion Details Under ACTT-1 Criteria

eFigure 2. Timing of Remdesivir and Corticosteroid Coadministration

eTable 1. Characteristics of Patients That Satisfied ACTT-1 Criteria, Before and After Propensity Score Matching

eTable 2. Characteristics of Remdesivir Patients Stratified by Corticosteroid Use

eTable 3. Adverse Events

eReferences.

This supplementary material has been provided by the authors to give readers additional information about their work.

© 2021 Garibaldi BT et al. *JAMA Network Open*.

eMethods

Time-dependent Propensity Score Matching

Since the timing of initial remdesivir administration was different and the remdesivir assignment was non-randomized, a time-dependent propensity score matching method was applied to undertake 1:1 propensity score matching for the treatment arm so that additional analyses could be performed on the matched patients. Propensity scores were calculated from a time-dependent Cox proportional hazards regression model using the time to the first receipt of remdesivir as the outcome, where the propensity score at a given hospitalization day is the hazard of exposure to remdesivir treatment at that day. The PH assumption was checked using the Schoenfeld residuals against the transformed time.[1-2]

Time-dependent covariates in records before the first remdesivir administration date (for treatment group patients) and records before right-censoring or last follow-up date (for control group patients) were used as predictors to obtain parameter estimates. Time-invariant (fixed) covariates included hospital admission race, age, sex, body mass index (BMI), Charlson comorbidity index (CCI), and code status (i.e. whether the patient had a “do not resuscitate” order). Time-dependent (varying) covariates included various clinical measures of disease severity, such as the ratio of measured oxygen saturation to fraction of inspired oxygen (SpO2/FiO2), systolic blood pressure (SBP), diastolic blood pressure (DBP), pulse, temperature, respiratory rate, and supplemental oxygen device (O2 device). SpO2/FiO2 was selected since it can be calculated without obtaining an arterial blood gas (not available on all patients). Other time-dependent variables included key laboratory test results, such as C-reactive protein (CRP), absolute lymphocyte count (ALC), platelets count, white blood cell count (WBC), hemoglobin (HGB), albumin, alanine aminotransferase (ALT), estimated Glomerular Filtration Rate (eGFR), and D-dimer.

Beginning from day zero, a sequential 1:1 greedy matching without replacement was conducted. Patients were included in the matching process only if their admission dates were later than the earliest admission date (April 27, 2020) of patients in the remdesivir group. A patient who first received remdesivir at a given day t of hospitalization was matched with those who did not, based on their propensity scores (hazard components) at day t. In addition, a time constraint was imposed so that a patient in the remdesivir group with k days of treatment, was forced to match a patient in the control group who stayed at least k days (5 days maximum) in the hospital since the matched day. This constraint removed patients from the control group who were healthy enough to be discharged in one or two days

© 2021 Garibaldi BT et al. *JAMA Network Open*.

from the matched day as those patients were unlikely to receive remdesivir treatment at the matched day if they were close to discharge.

Marginal Structural Cox Regression Model

In sub-analyses considering the effects of combination drug use, comparisons of patients who used combination of corticosteroids and remdesivir (n=185) with patients who used remdesivir alone (n=158) were conducted. Since the sample sizes for two groups were similar, and patients' exposure to corticosteroids was time-variant, instead of time-dependent propensity score matching, Marginal Structural Cox regression models were conducted to adjust for the non-randomized administration of corticosteroids, in the meantime, to analyze the effects of corticosteroids on outcomes of interests in patients who had exposure to remdesivir.[3] The same set of time-dependent covariates and time-invariant variables as in the matching models from all remdesivir patients were included in the model. The Inverse Probability Treatment Weighting (IPTW) method was applied for parameter estimation.

Outcome of Interest Analyses

The primary outcome of interest was time to clinical improvement from the treatment start of remdesivir, defined as discharge alive from the hospital without worsening of their WHO disease severity score or at least a two-point decrease in the WHO severity score during hospitalization within 28 days or max follow-up after the first treatment of remdesivir. Failure of clinical improvement was censored at max follow-up day or 28 days, whichever came first. Death was also censored at 28 days. The secondary outcome was time to death from the first remdesivir treatment day. Patients who were discharged alive were censored at 28 days.

Cox proportional-hazards regression models were applied to estimate the association between remdesivir treatment and outcomes of interests. A set of demographics, clinical variables and laboratory test results were included in Cox regression models based on clinical interest and knowledge. Time-invariant variables included race, age, sex, body mass index (BMI), Charlson comorbidity index (CCI), and code. Time dependent covariates included SpO2/FiO2 ratio, SBP, DBP, CRP, ALC, respiratory rate, temperature, pulse, WBC, HGB, Albumin, ALT, eGFR, and D-dimer. The adjusted hazard ratio of remdesivir treatment was estimated from the Cox regression model after controlling these covariates.

© 2021 Garibaldi BT et al. *JAMA Network Open*.

Missing Data Imputations

For the laboratory results, missing values were imputed using the last observation carried forward if the last observation was within three days of the missing data, otherwise, using multiple imputation by chained equations (MICE) with predictive mean matching method.[4]

Time to Clinical Improvement Using a 1-point Decrease in WHO severity score

We repeated the time to improvement analyses using a 1-point improvement in the WHO severity score and the results did not appreciably change. 239 (83.9%) remdesivir patients and 221 (77.5%) controls achieved clinical improvement before 28 days with a median time to clinical improvement of 4.0 days (IQR 3.0 – 7.0) and 5.0 days (IQR 3.0 – 9.0 days), respectively. In Cox proportional hazards models, remdesivir significantly shortened time to clinical improvement (aHR 1.28 [1.07; 1.55]). The results are sensitive to the requirement that controls be selected from among patients who remained hospitalized during the same period of treatment as their matched counterpart (up to 5 days). Although there is no qualitative change if the requirement is reduced to 4 days, the estimated aHR decreases to 1.07 [0.89;1.28] if the required period of hospitalization is lowered to 3 days or less. This is because 87 patients in the control group had their event (78 were discharged, and 9 achieved clinical improvement) within 4 days of matching compared to 26 patients (23 discharged and 3 improvement) in the treatment group.

© 2021 Garibaldi BT et al. *JAMA Network Open*.

eFigure 1. Exclusion Details under ACTT-1 Criteria

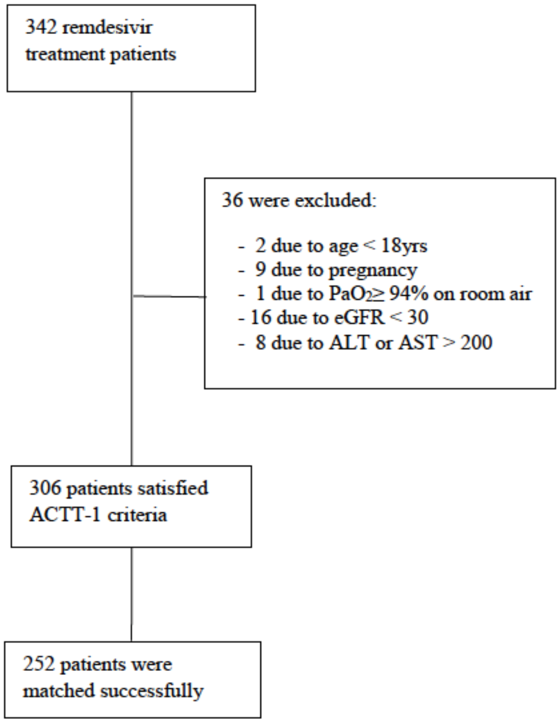

eFigure1 Legend. ACTT-1 Criteria: patients were excluded if age < 18 years, or pregnant, or $PaO_2 \geq 94\%$ on room air, or either alanine aminotransferase (ALT) or aspartate aminotransferase (AST) > 200, or estimated glomerular filtration rate (eGFR) < 30.

© 2021 Garibaldi BT et al. *JAMA Network Open*.

eFigure 2. Timing of Remdesivir and Corticosteroid Coadministration

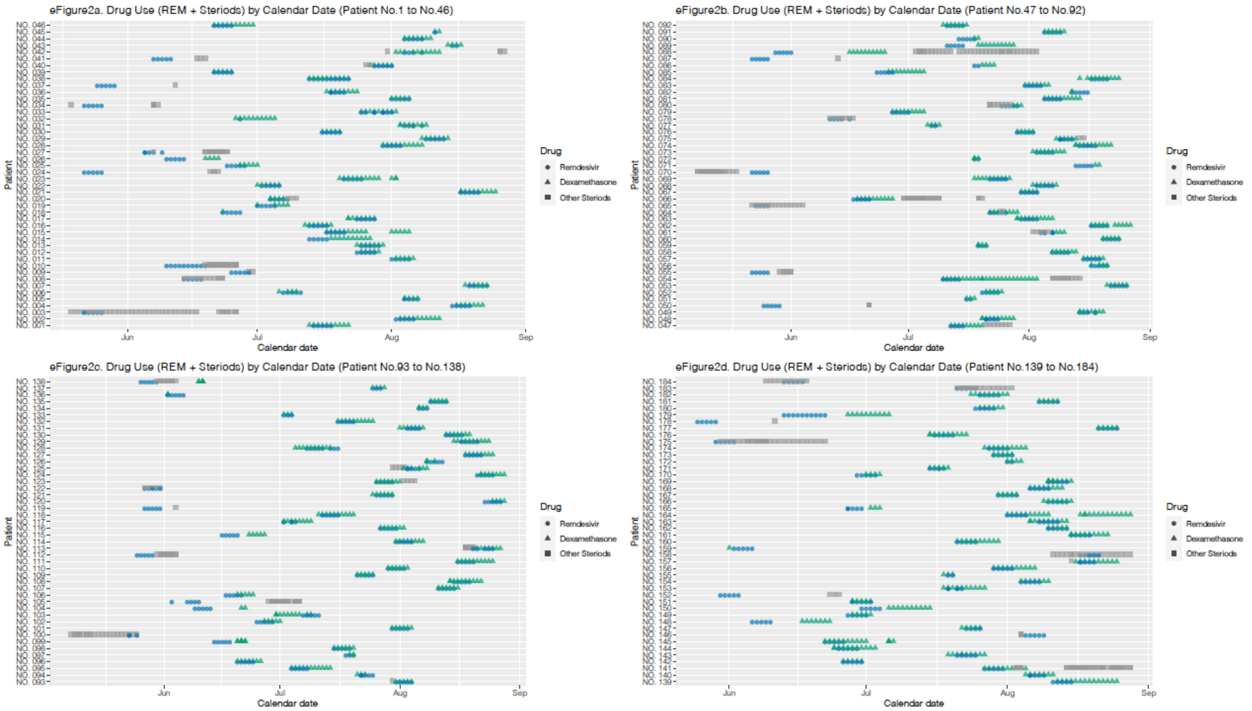

eFigure 2 Legend. The time-course for co-administration of remdesivir and corticosteroids is shown for the 185 patients who received both corticosteroids and remdesivir. The majority of patients received 6mg of dexamethasone daily as utilized in the RECOVERY trial.

© 2021 Garibaldi BT et al. *JAMA Network Open*.

eTable 1. Characteristics of Patients That Satisfied ACTT-1 Criteria, Before and After Propensity Score Matching

Characteristics	*All Remdesivir Patients Satisfied Criteria*† *(n = 306)*	Propensity Score – Matched Patients†		
		Matched Remdesivir (n=252)	*Matched Control (n=252)*	*Absolute Standardized Differences*
**Demographics:**				
Male	176 (57.5%)	147 (58.3%)	144 (57.1%)	0.024
Race Black	105 (34.3%)	77 (30.6%)	87 (34.5%)	0.085
Race Latinx	103 (33.7%)	88 (34.9%)	80 (31.7%)	0.067
Race White	62 (20.3%)	56 (22.2%)	55 (21.8%)	0.010
Race Others	36 (11.8%)	31 (12.3%)	30 (11.9%)	0.012
Age, Median (IQR)	60.5 (10)	62 (10)	61 (11.1)	0.039
BMI, Median (IQR)	31.1 (5.1)	30.1 (4.4)	30 (4.6)	0.073
**DNR/DNI, no. (%)**	53 (17.3%)	49 (19.4%)	61 (24.2%)	0.115
**O2 Devices, no. (%):**				
No Supplemental Oxygen	11 (3.6%)	11 (4.4%)	11 (4.4%)	0.000
Nasal Cannula or Face Mask	193 (63.1%)	171 (67.9%)	151 (59.9%)	0.166
High Flow Nasal Cannula	54 (17.6%)	32 (12.7%)	45 (17.9%)	0.144
Noninvasive Positive-Pressure Ventilation	4 (1.3%)	4 (1.6%)	5 (2%)	0.030
Mechanical Ventilator	44 (14.4%)	34 (13.5%)	37 (14.7%)	0.034
**Vital Signs, Mean (SD):**				
Temperature (°Celsius)	37.9 (0.9)	37.8 (0.8)	37.9 (0.9)	0.072
Pulse (beats per minute)	96 (18.4)	96.1 (19.1)	98.6 (18.3)	0.132
Systolic BP (mmHg)	105.6 (16.7)	106 (17)	106.4 (17.4)	0.024
Diastolic BP (mmHg)	58.2 (10.5)	58.9 (10.6)	58.2 (11.7)	0.064
SpO_2/FiO_2	334.4 (110.4)	345.4 (109.4)	336.7 (119.4)	0.076
**Laboratory Results, Mean (SD):**				
Estimated glomerular filtration rate	89.9 (27.2)	88 (25.9)	87.1 (28.2)	0.032
(ml/min)	11.7 (8.2)	11.8 (8.5)	11.9 (10)	0.020
C-reactive protein (mg/dL)	1.1 (0.7)	1.1 (0.7)	1 (0.5)	0.116
Absolute lymphocyte count (K cells/mm^3)	236.7 (107.4)	235.9 (105.5)	224.5 (96.3)	0.113
Platelet count (K cells/mm^3)	8.1 (6.1)	8.1 (6.5)	8.3 (4.6)	0.034
White blood cell count (K cells/mm^3)	12.2 (1.9)	12.2 (1.9)	12.1 (2.2)	0.039
Hemoglobin (g/dL)	3.1 (0.6)	3.2 (0.6)	3.2 (0.6)	0.035
Albumin (g/dL)	43.6 (31.8)	42 (30.8)	40.7 (33.6)	0.040
Alanine aminotransferase (U/L)	2.2 (4.5)	2.3 (4.7)	2.1 (4.1)	0.033
D-dimer (mg/L FEU)				
**Past Diagnoses, no. (%)**				
Hypertension	131 (42.8%)	110 (43.7%)	96 (38.1%)	0.113
Coronary Artery Disease	83 (27.1%)	75 (29.8%)	79 (31.3%)	0.034
Congestive heart failure	38 (12.4%)	32 (12.7%)	43 (17.1%)	0.123
Chronic kidney disease	17 (5.6%)	16 (6.3%)	11 (4.4%)	0.088
Diabetes	96 (31.4%)	83 (32.9%)	71 (28.2%)	0.104
Asthma	24 (7.8%)	19 (7.5%)	22 (8.7%)	0.044
COPD/Chronic Lung Disease	53 (17.3%)	43 (17.1%)	36 (14.3%)	0.076
Cancer	20 (6.5%)	19 (7.5%)	19 (7.5%)	0.000
Liver Disease	11 (3.6%)	11 (4.4%)	12 (4.8%)	0.019
AIDS/HIV	3 (1%)	2 (0.8%)	3 (1.2%)	0.040
Transplant	7 (2.3%)	7 (2.8%)	3 (1.2%)	0.114
Charlson Comorbidity Index:				
=0	128 (41.8%)	97 (38.5%)	105 (41.7%)	0.065
1-4	172 (56.2%)	150 (59.5%)	144 (57.1%)	0.048
>=5	6 (2%)	5 (2%)	3 (1.2%)	0.064
**Concomitant Medications, no. (%):**				
Hydroxychloroquine	1 (0.3%)	1 (0.4%)	5 (2%)	0.147
Azithromycin	137 (44.8%)	111 (44%)	113 (44.8%)	0.016
Dexamethasone	143 (46.7%)	122 (48.4%)	37 (14.7%)	0.779
Prednisone	22 (7.2%)	19 (7.5%)	13 (5.2%)	0.098
Methylprednisolone	17 (5.6%)	14 (5.6%)	23 (9.1%)	0.137
Hydrocortisone	9 (2.9%)	7 (2.8%)	16 (6.3%)	0.172
Heparin	269 (87.9%)	220 (87.3%)	214 (84.9%)	0.069

† Data shown is from the day of remdesivir treatment initiation

© 2021 Garibaldi BT et al. *JAMA Network Open*.

eTable 2. Characteristics of Remdesivir Patients Stratified by Corticosteroid Use

Characteristics	All Remdesivir Patients†		
	Rem Alone (n = 158)	Rem + Steroids (n = 184)	Absolute Standardized Differences
**Demographics:**			
Male	88 (55.7%)	101 (54.9%)	0.016
Race Black	55 (34.8%)	69 (37.5%)	0.056
Race Latinx	64 (40.5%)	50 (27.2%)	0.285
Race White	26 (16.5%)	40 (21.7%)	0.135
Race Others	13 (8.2%)	25 (13.6%)	0.173
Age, Median (IQR)	56 (12.2)	63 (10)	0.249
BMI, Median (IQR)	31.5 (5.3)	30.1 (3.4)	0.178
**DNR/DNI, no. (%)**	18 (11.4%)	43 (23.4%)	0.320
**O2 Devices, no. (%):**			
No Supplemental Oxygen	7 (4.4%)	9 (4.9%)	0.022
Nasal Cannula or Face Mask High Flow	108 (68.4%)	102 (55.4%)	0.268
Nasal Cannula	21 (13.3%)	39 (21.2%)	0.210
Noninvasive Positive-Pressure Ventilation	1 (0.6%)	4 (2.2%)	0.131
Mechanical Ventilator	21 (13.3%)	30 (16.3%)	0.085
**Vital Signs, Mean (SD):**			
Temperature (^{o}Celsius)	38 (0.8)	37.7 (0.9)	0.309
Pulse (beats per minute)	99 (19.9)	94.2 (17.8)	0.254
Systolic BP (mmHg)	105.2 (15.9)	105.7 (17.7)	0.032
Diastolic BP (mmHg)	58.1 (9.3)	57.9 (11.4)	0.016
SpO_2/FiO_2	347.2 (108.4)	323.8 (110.1)	0.215
**Laboratory Results, Mean (SD):**			
Estimated glomerular filtration rate (ml/min)	90.4 (31.9)	84.5 (30.1)	0.190
C-reactive protein (mg/dL)	12 (7.8)	11.4 (8.9)	0.075
Absolute lymphocyte count (K cells/mm^3)	1.2 (0.6)	1 (0.7)	0.217
Platelet count (K cells/mm^3)	235.6 (108.6)	234.9 (102.6)	0.006
White blood cell count (K cells/mm^3)	8 (7.7)	8.2 (4)	0.037
Hemoglobin (g/dL)	12 (2)	12.1 (2)	0.045
Albumin (g/dL)	3 (0.6)	3.2 (0.6)	0.324
Alanine aminotransferase (U/L)	43.3 (34.9)	45.7 (39.1)	0.063
D-dimer (mg/L FEU)	2.7 (5.4)	1.9 (3.8)	0.177
**Past Diagnoses, no. (%)**			
Hypertension	63 (39.9%)	89 (48.4%)	0.172
Coronary Artery Disease	34 (21.5%)	58 (31.5%)	0.228
Congestive heart failure	20 (12.7%)	25 (13.6%)	0.028
Chronic kidney disease	10 (6.3%)	18 (9.8%)	0.127
Diabetes	53 (33.5%)	58 (31.5%)	0.043
Asthma	11 (7%)	18 (9.8%)	0.102
COPD/Chronic Lung Disease	18 (11.4%)	41 (22.3%)	0.294
Cancer	12 (7.6%)	11 (6%)	0.064
Liver Disease	5 (3.2%)	8 (4.3%)	0.062
AIDS/HIV	0 (0%)	3 (1.6%)	0.182
Transplant	2 (1.3%)	6 (3.3%)	0.134
Charlson Comorbidity Index:			
=0	72 (45.6%)	70 (38%)	0.153
1-4	81 (51.3%)	110 (59.8%)	0.172
>=5	5 (3.2%)	4 (2.2%)	0.061
**Concomitant Medications, no. (%):**			
Hydroxychloroquine	1 (0.6%)	2 (1.1%)	0.049
Azithromycin	75 (47.5%)	78 (42.4%)	0.102
Dexamethasone	N/A	157 (85.3%)	N/A
Prednisone	N/A	27 (14.7%)	N/A
Methylprednisolone	N/A	20 (10.9%)	N/A
Hydrocortisone	N/A	12 (6.5%)	N/A
Heparin	140 (88.6%)	152 (82.6%)	0.172

† Data shown is from the day of Remdesivir treatment initiation

Rem denotes Remdesivir. Steroids denotes Corticosteroids (dexamethasone, prednisone, prednisolone, methylprednisolone or hydrocortisone)

© 2021 Garibaldi BT et al. *JAMA Network Open*.

eTable 3. Adverse Events

	Matched Remdesivir (n = 285)	Matched Control (n = 285)
**Before Matched Day or Drug Day, no. (%):**		
ALT or AST > 200 IU	5 (1.8%)	7 (2.6%)
bilirubin > 2 mg/dL	3 (1.1%)	4 (1.4%)
eGFR < 30 ml/min	3 (1.1%)	4 (1.4%)
**On/After Matched Day or Drug Day, no. (%):**		
ALT or AST > 200 IU	29 (10.2%)	29 (10.2%)
bilirubin > 2 mg/dL	10 (3.5%)	17 (6.0%)
eGFR < 30 ml/min	27 (9.5%)	58 (20.4%)
**Anytime in hospital, no. (%):**		
ALT or AST > 200 IU	32 (11.2%)	36 (12.6%)
bilirubin > 2 mg/dL	11 (3.9%)	18 (6.3%)
eGFR < 30 ml/min	31 (10.9%)	71 (24.9%)

eReferences

1. Lu B. Propensity score matching with time-dependent covariates. Biometrics 2005;61:721-8.
2. Xie Y, Bowe B, Li T, Xian H, Yan Y, Al-Aly Z. Risk of death among users of Proton Pump Inhibitors: a longitudinal observational cohort study of United States veterans. BMJ Open 2017;7:e015735.
3. Hernán M Á, Brumback B, Robins J M. Marginal structural models to estimate the causal effect of zidovudine on the survival of HIV-positive men[J]. Epidemiology, 2000: 561-570.
4. van Buuren S, Groothuis-Oudshoorn K. mice: Multivariate imputation by chained equations in R. J Stat Software 2011;45:1-67.

© 2021 Garibaldi BT et al. *JAMA Network Open*.
